# Therapeutic applications of circadian rhythms for the cardiovascular system

**DOI:** 10.3389/fphar.2015.00077

**Published:** 2015-04-17

**Authors:** Elena V. Tsimakouridze, Faisal J. Alibhai, Tami A. Martino

**Affiliations:** Cardiovascular Research Group, Department of Biomedical Sciences, University of GuelphGuelph, ON, Canada

**Keywords:** chronotherapy, circadian, diurnal, biomarkers, cardiovascular disease

## Abstract

The cardiovascular system exhibits dramatic time-of-day dependent rhythms, for example the diurnal variation of heart rate, blood pressure, and timing of onset of adverse cardiovascular events such as heart attack and sudden cardiac death. Over the past decade, the circadian clock mechanism has emerged as a crucial factor regulating these daily fluctuations. Most recently, these studies have led to a growing clinical appreciation that targeting circadian biology offers a novel therapeutic approach toward cardiovascular (and other) diseases. Here we describe leading-edge therapeutic applications of circadian biology including (1) timing of therapy to maximize efficacy in treating heart disease (chronotherapy); (2) novel biomarkers discovered by testing for genomic, proteomic, metabolomic, or other factors at different times of day and night (chronobiomarkers); and (3) novel pharmacologic compounds that target the circadian mechanism with potential clinical applications (new chronobiology drugs). Cardiovascular disease remains a leading cause of death worldwide and new approaches in the management and treatment of heart disease are clearly warranted and can benefit patients clinically.

## Introduction

Cardiovascular disease is the leading cause of death worldwide (Public Health Agency of Canada, 2009; World Health Organization [WHO], 2011; Mozaffarian et al., 2014; Townsend et al., 2014). Available therapies have had only limited success improving long-term survival of patients. In recent years there have been a flurry of studies demonstrating time-of-day variations in drug toxicity and efficacy (reviewed in Smolensky and D’Alonzo, 1988; Smolensky and Peppas, 2007), daily cardiovascular gene and protein expression (reviewed in Martino and Sole, 2009; Durgan and Young, 2010; Paschos and FitzGerald, 2010), and there are reports of new pharmacological compounds targeting the circadian mechanism (reviewed in Chen et al., 2013; Kojetin and Burris, 2014). These have led to novel opportunities to investigate and apply the important field of chronobiology on clinical cardiology, and medicine in general.

**FIGURE 1:**
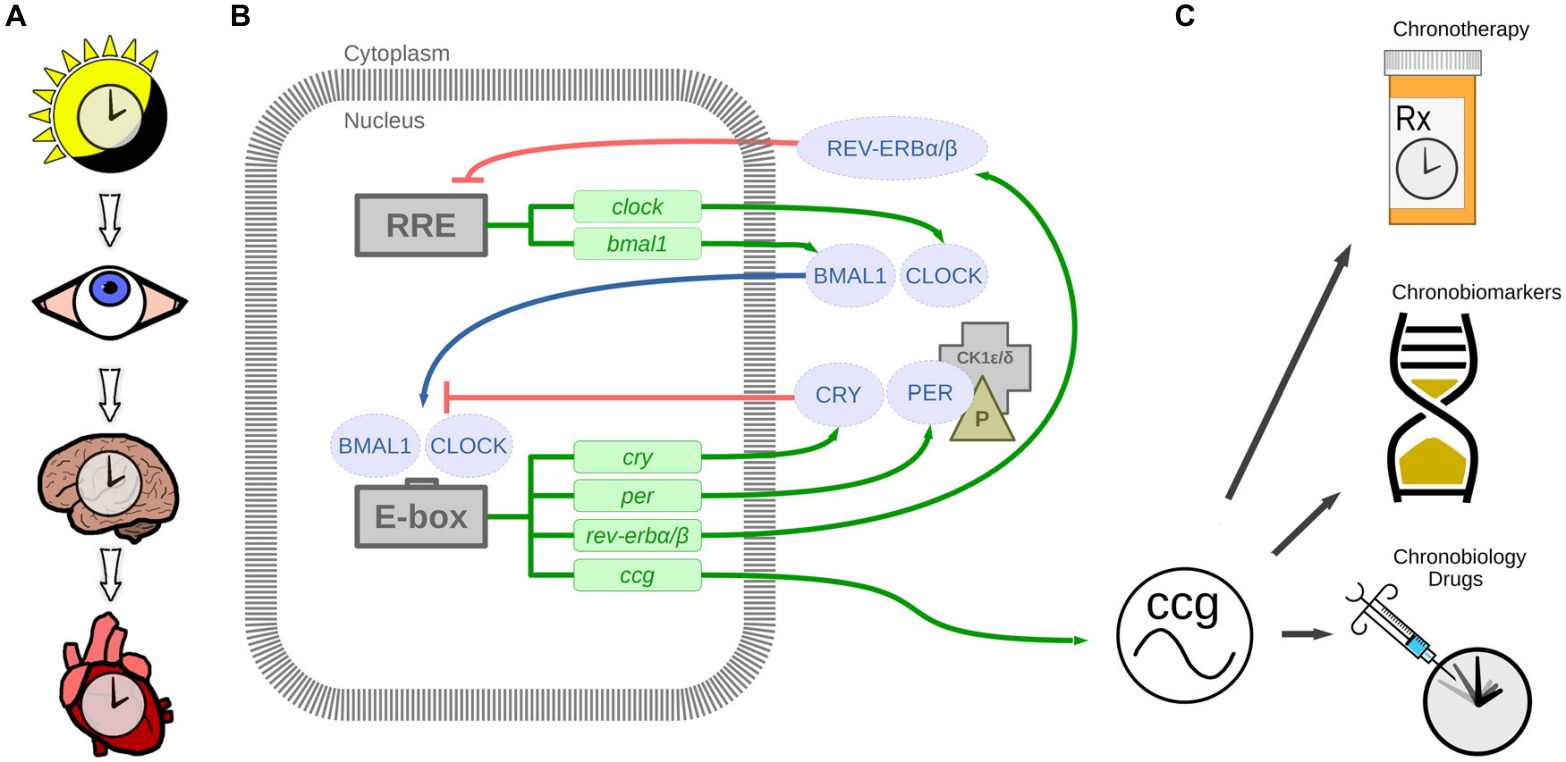
**The circadian timing system. (A)** Light stimulus is relayed by the eye to the suprachiasmatic nucleus in the brain, which in turn synchronizes the heart and other organ clocks to the day and night environment. **(B)** These signals entrain the molecular clock mechanism, which keeps 24-h time in tissues and cells via transcription-translation feedback loops. BMAL1 and CLOCK are transcribed and translated. BMAL1 and CLOCK heterodimers bind to E-box enhancer elements to promote transcription of *cryptochrome* (CRY), *period* (PER), nuclear receptor subfamily 1, group D, member 1/2 (rev-erbα/β; nr1d1/2), and other clock controlled genes (ccg). Proteins CRY and PER are phosphorylated by casein kinase 1δ/𝜀 (CK1δ/𝜀) in the cytoplasm, which translocate to the nucleus to repress CLOCK and BMAL1 mediated transcription. Additional loops exist whereby REV-ERBα/β negatively regulates *bmal1* transcription by binding to RRE (REV-ERB/retinoic acid receptor-related orphan receptor (ROR) response element). This mechanism regulates 24-h transcription of clock controlled genes which in play a crucial role in diurnal cardiovascular physiology. **(C)** Therapeutic applications of circadian rhythms include chronotherapy by timing treatment to daily rhythmic processes, chronobiomarkers of differing rhythmic profiles between health and disease, and new chronobiology drugs targeting the circadian clock mechanism.

The underlying foundation for cardiovascular chronotherapy stems from observations that biological processes in humans (and other mammals) exhibit 24-h daily rhythms, and these are controlled by molecular circadian clocks in the brain, heart, and other organs (**Figures 1A,B**). There are many excellent reviews on the circadian system (reviewed in Hastings et al., 2003; Roenneberg and Merrow, 2005; Dardente and Cermakian, 2007; Mohawk et al., 2012). Cardiovascular physiology appears to follow a rhythm as well; heart rate (HR), blood pressure (BP), and cardiac contractility all peak in the wake hours and reach a nadir during sleep (reviewed in Martino and Sole, 2009; Durgan and Young, 2010; Paschos and FitzGerald, 2010). Indeed, many cardiovascular functions that oscillate over the 24-h period are influenced by the circadian clock mechanism as well as daily fluctuations in the neurohormonal milieu (reviewed in Bray and Young, 2008; Sole and Martino, 2009; Gamble et al., 2014). Timing of onset of cardiac pathologies also follows a rhythm (e.g., onset of myocardial infarction [MI, or heart attack; Muller et al., 1985), and sudden cardiac death (Muller et al., 1987)]. These time-of-day variations in cardiovascular physiology and pathophysiology have led to a growing clinical appreciation that endogenous circadian rhythms may be an important factor to consider in treating disease. Here, we review the current knowledge regarding therapeutic applications of circadian rhythms for the cardiovascular system (**Figure 1C**), specifically (1) timing of therapy (chronotherapy), (2) circadian biomarkers (chronobiomarkers), and (3) how modifiers of the circadian clock mechanism may be useful in the treatment of heart disease.

## Chronotherapy

### Rationale

Chronotherapy is an important therapeutic application of circadian rhythms for the cardiovascular system. The rationale for chronotherapy is that it offers translational benefit by considering factors such as the underlying circadian rhythms in drug pharmacology, specifically pharmacokinetics (i.e., drug absorption, distribution, metabolism, and excretion) and pharmacodynamics (i.e., affinity and specificity for target receptor binding, downstream intracellular signaling). Chronotherapy also takes into account the patients’ underlying physiology and disease pathology (reviewed in Labrecque and Belanger, 1991; Reinberg, 1991; Paschos et al., 2010; Musiek and Fitzgerald, 2013). That the majority of the best-selling drugs and World Health Organization essential medicines target the products of circadian genes provides a mechanistic basis for understanding chronotherapy (Zhang et al., 2014), and provides further support for the clinical application of chronotherapy. Specific examples applied to the treatment of cardiovascular disease are discussed in further detail below. We also created a blog featuring published chronotherapy studies for cardiovascular and other diseases^1^.

### Chronotherapy Decreases Adverse Cardiovascular Remodeling

In our recent pre-clinical study in mice, we showed that chronotherapy can have direct benefits on the heart in cardiovascular disease models (Martino et al., 2011). Mice with pressure-overload induced cardiac hypertrophy were administered the short-acting angiotensin converting enzyme inhibitor (ACEi) captopril at either sleep-time or wake-time. We found that only sleep-time administration improves cardiac function, and reduces cardiac remodeling, as compared to wake-time captopril and placebo-treated animals. Mechanistically, captopril given at sleep-time appears to target the peak in the renin-angiotensin-system gene profiles in the heart (Martino et al., 2011). Thus this study demonstrates the direct beneficial effects of chronotherapy for cardiac hypertrophy in the murine model. The important clinical implications are that ACEis given at bedtime can benefit myocardial remodeling in hypertensive patients, or after MI, or in congestive heart failure. Indeed, clinically, ACEis are one of the most commonly prescribed drugs given to hypertensive patients and also for ischemic heart disease (Pfeffer et al., 1992; AIRE, 1993; Ambrosioni et al., 1995; Kober et al., 1995; Yusuf et al., 2000; Fox and EURopean trial On reduction of cardiac events with Perindopril in stable coronary Artery disease Investigators, 2003; Nissen et al., 2004).

### Chronotherapy Benefits Daily BP and HR Rhythms

Diurnal BP rhythms are an important part of healthy cardiovascular physiology, and thus are also a key target for chronotherapeutic strategies. Indeed, it is well-known that daily BP profiles are characterized by a dramatic BP surge that occurs around the time of wakening, followed by a progressive fall (∼10%) to reach a nadir during sleep (Floras et al., 1978; Millar-Craig et al., 1978). Conversely, loss of the nocturnal BP fall (non-dipper profile) adversely affects the heart (Verdecchia et al., 1990; Ohkubo et al., 2002; Dolan et al., 2005; Fagard et al., 2009), and chronotherapy to improve the nocturnal BP profile is beneficial. There are many studies that take a chronotherapeutic approach to regulate 24-h BP profiles in hypertensive patients. This includes treatment with ACEis, angiotensin receptor blockers (ARBs), β-blockers, acetylsalicylic acid (aspirin), and combination therapies at specific times of day or night. These studies are summarized in **Table 1**.

**Table 1:** The benefits of chronotherapy for blood pressure (BP) in patients with mild to moderate hypertension.

Drug (dose)	Study design (n)	Chronotherapeutic benefit	Reference
**Angiotensin converting enzyme inhibitor (ACEi)**	
**Quinapril** (20 mg/day for 4 weeks)	Double-blind cross-over (18)	Evening quinapril more effectively decreased nighttime BP and 24 h BP profile compared to morning treatment	Palatini (1992)
**En alapril** (10 mg/day for 3 weeks)	Randomized cross-over (10)	Evening enalapril caused a greater reduction in nocturnal BP as compared to morning administration	Witte et al. (1993)
**Lisinopril** (20 mg/day for 2 months)	Randomized cross-over (40)	Evening lisinopril resulted in a largest reduction in morning BP (6 AM–11 AM) as compared to morning and afternoon treatment	Macchiarulo et al. (1999)
**Ramipril** (5 mg/day for 6 weeks)	PROBE^a^ multicenter (115)	Bedtime ramipril led to the largest decrease in sleep-time BP and increased the number of patients with controlled 24 h ambulatory BP	Hermida and Ayala (2009)
**Spirapril** (6 mg/day for 12 weeks)	Randomized, open-label, parallel group, blinded endpoint (165)	Bedtime spirapril more effectively decreased the nocturnal BP and increased the proportion of patients with controlled 24 h ambulatory BP	Hermida et al. (2010a)
**Angiotensin receptor blocker (ARB)**	
**Valsartan** (160 mg/day for 3 months)	PROBE^a^ non-dipper (148)	Bedtime valsartan further decreased nocturnal BP mean and led to a greater proportion of patients with dipper profiles and controlled BP over 24 h as compared to treatment upon awakening	Hermida et al. (2005b)
**Telmisartan** (80 mg/day for 12 weeks)	PROBE^a^ (215)	Bedtime telmisartan further decreased sleep-time BP and increased the number of patients with dipper profiles as compared to morning administration	Hermida et al. (2007)
**Olmesartan** (20 mg/day for 3 months)	PROBE^a^ (123)	Bedtime olmesartan resulted in the largest reduction in nocturnal BP mean and decreased prevalence of non-dipping from baseline as compared to the morning dose	Hermida et al. (2009)
**β-Blocker**	
**Nebivolol** (5 mg/day for 1 week; titrated to 10 mg/day for 2 weeks)	Single-center, prospective, randomized, double-blind, placebo controlled, cross-over (38)	Evening but not morning, nebivolol significantly decreased morning preawakening systolic BP from baseline	Acelajado et al. (2012)
**Non-steroidal anti-inflammatory drugs**	
**Acetylsalicylic acid** (100 mg/day for 3 months)	PROBE^a^ (328)	Only bedtime administration reduced the 24 h BP mean, but not treatment upon awakening	Hermida et al. (2005a)
**Combination therapy**	
**Amlodipine** (2.5–10 mg) **plus Olmesartan** (20–40 mg) (for 8 weeks)	Randomized, open-label, crossover (31)	Evening treatment significantly decreased the morning BP surge and decreased nocturnal BP in non-dippers as compared with morning treatment	Hoshino et al. (2010)
**Valsartan** (160 mg/day) **plus Hydrochlo-rothiazide** (12 mg/day) (for 12 weeks)	PROBE,^a^ (204)	Bedtime dose more effectively reduced sleep-time systolic BP mean and increased the proportion of patients with controlled sleep-time BP as compared to treatment upon awakening	Hermida et al. (2011)
**Valsartan** (160 mg/day) **plus Amlodipine** (5 mg/day) (for 12 weeks)	PROBE (203)	Bedtime administration more efficiently decreased the 48h BP mean, lowered sleep-time BP, and had the largest percentage of patients with controlled BP over 24 h compared to morning administration	Hermida et al. (2010b)

Prospective, randomized, open-label, parallel group, blinded endpoint (PROBE).

Intriguingly, HR also exhibits a rhythm that peaks in the day and is lowest at night (Clarke et al., 1976). The effects of chronotherapy on HR are not as well investigated as with BP profiles, however, several studies have indicated a time-of-day influence of β-blockers on HR. (1) In healthy subjects, the β-blocker propanolol exhibits a significantly faster time to peak effect on HR if taken in the morning (8 A.M.) as compared to late at night (2 A.M; Langner and Lemmer, 1988). (2) The suppressive effect of propranolol on the rise in HR during exercise is significantly greater if the drug is taken in the morning versus at night (Fujimura et al., 1990). (3) In patients with stable coronary disease, myocardial ischemic episodes associated with HR increases are more likely to occur during the day time than at night; propranolol reduces the proportion of these daily HR-related episodes (Andrews et al., 1993). (4) In hypertensive patients, the β-blocker bisoprolol reduces the 24-h ambulatory HR if the drug is taken in the morning (Mengden et al., 1992). (5) Lastly, experimental studies in rodents help confirm that HR is differentially influenced by some β-blockers depending on the time of drug application; propanolol causes a near maximum decrease in HR when given in the light period (rodent sleep time) as compared to the dark period (rodent wake time; Lemmer et al., 1985). Collectively these findings illustrate the importance of maintaining daily BP and HR profiles, and the clinical applicability of chronotherapy to benefit cardiovascular physiology.

### Aspirin Chronotherapy and Timing of Acute Cardiovascular Events

In an exciting recent chronotherapy study, it was found that evening administration of low-dose aspirin reduces morning platelet reactivity, via COX-1 dependent pathways, as compared with taking aspirin upon awakening (Bonten et al., 2014). This finding is consistent with earlier reports of a circadian rhythm in platelet surface markers (Scheer et al., 2011), and in platelet aggregability (Andrews et al., 1996). Collectively these studies are clinically important because acute cardiovascular events (e.g., MI) are most likely to occur in the early morning hours vs. other times of day or night (Muller et al., 1985), and platelet reactivity likely contributes to this early morning peak. Thus it is postulated that aspirin chronotherapy taken at bedtime instead of on awakening, as a preventative measure in healthy subjects and by patients with cardiovascular disease, can reduce the incidence of adverse cardiac events during the high-risk morning hours (Bonten et al., 2014). That daily low-dose aspirin reduces the peak frequency of MIs in the morning and overall risk across the 24-h cycle (Ridker et al., 1990), provides further support for this notion.

It is worth noting that several factors important for thrombosis and fibrinolysis in MI, in addition to platelet reactivity and cycling, also exhibit daily rhythms and could provide additional targets for chronotherapy for treatment of acute cardiovascular events. These factors include plasminogen activator inhibitor-1 (PAI-1 a key inhibitor of fibrinolysis; Angleton et al., 1989; Scheer and Shea, 2013), tissue factor pathway inhibitor and factor VII (Pinotti et al., 2005), and plasma fibrinogen (Bremner et al., 2000). Moreover, several experimental rodent studies mechanistically link these coagulation pathways directly to the circadian clock mechanism. That is, transcription of the anti-coagulant factor thrombomodulin is regulated by the mechanism factors CLOCK and BMAL2 heterodimers (Takeda et al., 2007), and PAI-1 transcription is regulated by CLOCK and BMAL proteins (Schoenhard et al., 2003). Endothelial responses to vascular injury also appear to be regulated by the clock mechanism (Westgate et al., 2008). In terms of clinical translation, time-of-day variation in the efficacy of thrombolytic therapy in MI has been reported, which shows a marked early morning resistance and significantly better results later in the day (Reisin et al., 2004). Taken together, these and earlier studies provide support for cardiovascular chronotherapy to limit the pathogenesis and improve treatment following the onset of acute cardiovascular events.

### Nocturnal Hemodialysis (NHD) Benefits Cardiovascular Disease

Cardiovascular disease is a significant cause of death in patients with end-stage renal disease (Harnett et al., 1995; Collins et al., 2007), and left ventricular hypertrophy contributes to the high mortality rates in patients given conventional daytime hemodialysis (CHD) treatment (Harnett et al., 1994). Intriguingly, NHD, renal replacement therapy during sleep) offers better BP control (Pierratos et al., 1998; Raj et al., 1999), and is accompanied by regression of left ventricular hypertrophy (Chan et al., 2002), as compared to patients given conventional daytime therapy. In addition to decreasing the nighttime BP, NHD also decreases 24-h mean arterial BP compared to CHD (Chan et al., 2003). These findings of a chronotherapeutic benefit are further corroborated by a randomized controlled clinical trial demonstrating that frequent NHD improves systemic BP and reduces left ventricular mass compared with CHD (Culleton et al., 2007). Mechanistically, the beneficial effects of NHD are associated with changes in myocardial mechanics in patients, and experimentally correlated with unique cardiac gene expression signatures in rodent studies *in vivo* (Chan et al., 2012). These studies demonstrate chronotherapeutic benefit for the heart, in patients with end-stage renal disease, by chronotherapeutically converting from CHD to NHD treatment.

### Nocturnal Therapy for Obstructive Sleep Apnea Benefits the Heart

Obstructive sleep apnea (OSA) is a common sleep disorder, with cardiovascular consequences (e.g., through increased sympathetic activation, etc. as has been well reviewed in Bradley and Floras, 2003; Somers et al., 2008; Bradley and Floras, 2009; Kasai and Bradley, 2011; Ayas et al., 2014; Floras, 2014). OSA is a target for chronotherapy, as several studies have revealed that sleep time treatment with continuous positive airway pressure (CPAP) attenuates some of the adverse effects on the cardiovascular system. For example, CPAP therapy decreases the risk of non-fatal and fatal adverse cardiovascular events in severe OSA patients (apnea-hypopnea index >30 h) as compared to untreated patients, as demonstrated in a 10 years long term follow-up study (Marin et al., 2005). In another study, it was shown that CPAP therapy improves ejection fraction, lowers systolic BP, and reduces HR in heart failure patients with OSA (Kaneko et al., 2003). Also, CPAP treatment decreases cardiovascular-related deaths in OSA patients, as compared to an untreated OSA group, as was demonstrated over a follow-up period of 7.5 years (Doherty et al., 2005). Thus these studies underscore the notion that time-of-day therapies, such as nocturnal CPAP treatment, benefits cardiovascular physiology, and reduces pathophysiology in patients with OSA.

## Chronobiomarkers

### Definition

A second area for therapeutic application of circadian rhythms is in the development of time-of-day biomarkers for heart disease. The National Institutes of Health defines biomarkers as *“a characteristic that is objectively measured and evaluated as an indicator of normal biological processes, pathogenic processes, or pharmacologic responses to a therapeutic intervention”* (Biomarker Definitions Working Group, 2001). Classic biomarkers of cardiovascular disease relate to patient state (e.g., lifestyle risk factor profiles such as diet, exercise, and smoking) or biological processes (e.g., molecular gene and protein levels; reviewed in Jaffe et al., 2006; Maisel et al., 2006; Pletcher and Pignone, 2011). However, in contrast to these classic biomarkers which are measured during the daytime, chronobiomarkers provide a novel approach because clinical sampling is done at different times of day or night. Thus chronobiomarkers (unlike classic biomarkers) take into consideration the time-of-day rhythms important for body physiology and molecular processes. It is worth noting that timing of sampling is also relevant to translational research, since experiments on rodents are routinely performed during the working day when the animals are in their sleep period (rodents are nocturnal) with the intent of comparison to the human daytime. Sampling tissues and detecting biomarkers at different times across the day and night cycle can allow for better correlation with humans. New frontiers investigating molecular chronobiomarkers, with application to the clinical setting, are described below.

### Genomic Chronobiomarkers

Genomic chronobiomarkers are the most identifiable type of biomarker because the circadian clock mechanism is transcriptional in nature. That is, many labs have shown that the circadian mechanism underlies gene expression in the heart (and other) organs, and thus investigating how these gene patterns change in heart disease could lead to *de novo* chronobiomarker discoveries. The first large scale study examining rhythmic gene expression in the heart was by Storch et al. (2002), and revealed that ∼8*%* of genes (mRNA) in the murine heart exhibit circadian variations by microarray and bioinformatics analyses. Of note, this study was done under circadian (constant dark) conditions to elucidate clock controlled genes. However, since humans (and clinical medicine) exist in a 24-h light and dark and not circadian environment, we also demonstrated that ∼13*%* of murine cardiac genes (mRNA) exhibit rhythmic expression under normal day and night cycles, by microarray and COSOPT bioinformatics analyses (Martino et al., 2004). Most recently rhythmic mRNA profiles have also been shown in human heart tissue for the core clock genes (per1, per2, and bmal1; Leibetseder et al., 2009).

Interestingly, chromatin remodelers play a role in orchestrating time-of-day gene expression, by regulating rhythms in the epigenome (reviewed in Aguilar-Arnal and Sassone-Corsi, 2014), such as the histone deactylases termed silent information regulator 1 (SIRT1; Nakahata et al., 2008), and histone deacetylase 3 (HDAC3; Alenghat et al., 2008), and the histone methyltransferase termed mixed lineage leukemia 1 (MLL1; Katada and Sassone-Corsi, 2010). These are recruited to the promoters of clock controlled genes in a circadian manner, and rhythmic expression of clock controlled genes is altered in the absence of these chromatin modifiers (Alenghat et al., 2008; Nakahata et al., 2008; Katada and Sassone-Corsi, 2010). Moreover, the epigenetic markers of histone acetylation and methylation also exhibit rhythmic oscillations over 24 h (Etchegaray et al., 2003; Vollmers et al., 2012). In terms of therapeutic potential, pharmacological modulation with SIRT1 activators reduces histone acetylation and decreases the amplitude of circadian gene expression in mice (Bellet et al., 2013).

Since rhythmic gene expression underlies the vital cardiac processes, we also investigated whether time-of-day gene expression signatures could be utilized as *de novo* biomarkers of heart disease (i.e., chronobiomarkers). In a proof-of-concept study, we identified 300 mRNA chronobiomarkers, using a murine model of cardiac hypertrophy (transaortic constriction, TAC), microarrays, and a novel bioinformatics algorithm termed Delta Gene (Tsimakouridze et al., 2012). For example, the mitochondrial metabolism genes uncoupling protein 3 (Ucp3) and pyruvate dehydrogenase kinase 4 (Pdk4) exhibit significantly increased expression in TAC hearts in the light period (animals asleep) but not dark period (animals awake). Conversely, the apoptosis pathway gene BCL2/adenovirus E1B interacting protein 3 (Bnip3) exhibits increased expression in the dark. Moreover, we further demonstrated that day/night gene rhythms change over the course of the disease, and that later profiles can be predictive of heart failure. For example, decreased sleep-time expression of Ucp3 and increased wake-time expression of Bnip3 are simultaneously observed with progression to heart failure. (Tsimakouridze et al., 2012). Further optimization for clinical translation in heart disease would of course need to be considered, such as blood sampling instead of tissue, and the development of gene chips targeting specific disease profiles. Nevertheless, these early studies demonstrate the novelty and feasibility of such an approach, for genomic chronobiomarkers with application to clinical molecular diagnostics.

### Proteomic Chronobiomarkers

A second approach is to characterize the proteomic chronobiomarkers instead of the genetic markers. This is important because it is the proteins, and not the mRNA, that underlie many crucial biological processes in health and disease. In support of this approach, we demonstrated that ∼8% of the murine cardiac proteome exhibits significant changes in abundance over the 24-h day and night cycle, by using 2-dimensional difference in gel electrophoresis and liquid chromatography mass spectrometry (Podobed et al., 2012, 2014). Moreover, a role for the circadian clock mechanism is indicated in regulating time-of-day protein abundance, as differences in protein profiles are observed in the hearts of cardiomyocyte-specific clock mutant mice (Podobed et al., 2014). This includes many rate limiting enzymes important for key metabolic pathways in the heart (Podobed et al., 2014). As a proof-of-concept for application to heart disease, we demonstrated that protein chronobiomarkers have characteristic disease signatures in our murine model of TAC-induced cardiac hypertrophy (Podobed et al., 2012, 2014; Tsimakouridze et al., 2012). It is worth noting that although our studies report day/night protein signatures of heart disease, these studies rely on sampling directly from the heart tissue. For routine biomarker testing a more minimally invasive technique would need to be developed, such as detecting time-of-day protein biomarker signatures in the blood. To demonstrate the feasibility of less invasive testing, we showed time-of-day *de novo* chronobiomarkers in murine blood plasma samples, using surface-enhanced laser desorption/ionization mass spectrometry (Martino et al., 2007). In terms of translation, one interesting example illustrating the clinical potential of time-of-day biomarkers in heart disease comes from studies by Dominguez-Rodriguez et al. (2006), who show that nighttime serum melatonin levels are predictive of a subsequent adverse cardiovascular event in patients with ST-segment elevation MI. Thus taken together, these studies demonstrate significant clinical potential for protein chronobiomarkers for the diagnosis, prognosis, and personalized treatment of heart disease.

### Metabolomic Chronobiomarkers

The circadian clock regulates metabolism in the body (Turek et al., 2005; Paschos et al., 2012) and in the heart (reviewed in Young, 2006; Durgan and Young, 2010) and thus there is significant opportunity to investigate the circadian metabolome for chronobiomarkers of health and disease. For example, the liver metabolome exhibits rhythmic oscillations and disrupting the circadian clock mechanism alters these profiles (Eckel-Mahan et al., 2012). In another study in humans, it was demonstrated that ∼15% of metabolites in plasma and saliva samples are rhythmic and under circadian control (Dallmann et al., 2012). One clinical application is in the measurement of internal body time-of-day, which may be exploited to maximize efficacy and minimize toxicity of drugs therapies (e.g., for chronotherapy; Ueda et al., 2004). In this regard, the Ueda group designed a molecular-timetable of the murine blood metabolome, quantifying hundreds of clock controlled metabolites, using a liquid chromatography mass spectrometry approach (Minami et al., 2009). This same group subsequently applied their molecular metabolite timetable concept to successfully estimate internal body time in humans (Kasukawa et al., 2012). The CircadiOmics website provides a consolidated model that integrates these metabolomic data with genomics and proteomics, to better understand time-of-day coordination of physiology/pathophysiology (Patel et al., 2012). Indeed, taken together these data reveal the convenience and feasibility of adopting time-of-day testing for clinical use. It is tempting to speculate that additional “-omics” approaches, such as lipidomics or breathomics, could also be developed in the future as valuable clinical tools for personalized medicine.

## New Frontiers for Chronobiology Drugs

Recently, there has been a new focus on the creation of pharmacological compounds designed to target the REV-ERB and ROR nuclear receptors in the circadian mechanism, with clinical applications (reviewed in Kojetin and Burris, 2014). For example, administering REV-ERB agonists to mice alters their circadian behavior and hypothalamic gene expression, leading to the notion that these drugs may be useful in the treatment of metabolic disorders (Solt et al., 2012). Since REV-ERB also plays a key role in regulating mitochondrial content and the oxidative capacity of skeletal muscle, it is postulated that pharmacologic activation of REV-ERB may also be used to treat skeletal muscle diseases (Woldt et al., 2013). Moreover, it was recently shown that REV-ERB agonists can regulate sleep architecture and emotion in mice, and thus they may be useful in the treatment of sleep disorders and anxiety (Banerjee et al., 2014). There are new pharmacological agents that modulate other components of the circadian clock mechanism as well (e.g., reviewed in Chen et al., 2013); some of these hold considerable promise for offsetting the adverse effects of shift work (e.g., Walton et al., 2009; Meng et al., 2010; Pilorz et al., 2014). Most recently it was demonstrated that human peripheral blood mononuclear cell clocks are entrained by glucocorticoids, and that pharmacologic treatment directed at these peripheral targets could also help counteract the deleterious effects of shift work (Cuesta et al., 2014). Although the new chronobiology drugs have not yet been examined in heart disease, it is tempting to speculate that they may be useful, especially in light of their influences on muscle metabolism, on sleep, and on circadian phase, that they may benefit cardiovascular physiology and pathophysiology.

## Conclusions and future directions

In terms of future directions in basic science, use of murine transgenic models and pharmacologic approaches will undoubtedly provide new pre-clinical insights into how targeting the circadian mechanism can contribute to the diagnosis and management of heart disease. In terms of clinical chronotherapy, the US public clinical trials database (ClinicalTrials.gov., 2015) already lists seven studies when the search term “cardiovascular chronotherapy” is used, and 18 studies for “chronotherapy” in general, attesting to the clinical promise that chronotherapeutic treatments may hold. There are also significant opportunities to discover *de novo* chronobiomarker tests, for product development by biotechnology sectors, and for establishing routine applications in chronobiology, and sleep clinics. Thus therapeutic consideration of circadian rhythms for the cardiovascular system is an exciting new area with significant clinical potential.

## Conflict of Interest Statement

The authors declare that the research was conducted in the absence of any commercial or financial relationships that could be construed as a potential conflict of interest.

## Footnotes

1 http://chronobioapp.blogspot.ca/
